# Audit Of Psychotropic Prescribing in the Crisis Team at Fieldhead Hospital According to NICE Guidelines

**DOI:** 10.1192/bjo.2022.484

**Published:** 2022-06-20

**Authors:** Shumaila Shahbaz, Maya Garside, Tim Rajanna

**Affiliations:** South West Yorkshire Partnership NHS Foundation Trust, Wakefield, United Kingdom

## Abstract

**Aims:**

To ensure that psychotropic prescribing and monitoring in the Crisis Team is compliant with NICE guidelines and to provide excellent patient care and to practice medicine safely.

**Methods:**

Medication prescribing should be a collaborative decision by the service user and the prescriber. This allows patients to have autonomy to decide their treatment plan. NICE provides guidelines for prescribing medication which includes baseline investigations, reviews of treatment including side effects, and physical health monitoring.

We selected 50 admitted patients for the audit from April 2021 until September 2021, who were prescribed psychotropic medications. We used medication cards and electronic patients’ records (System One). Our exclusion criteria were the 72-hour post-discharge follow-up from the inpatient ward.

The audit standards included as follows: age, gender, the indication, the start of medications, dose, within BNF limits, discussion, consent from the patient, comorbidities, physical health monitoring, response to treatment, monitoring of side effects, and other important information.

**Results:**

100% results for indication, dosage, discussion with the patient, and side effects monitoring.

We had promising results for benefits from the treatment (46 out of 50 patients responded to treatment) and 4/50 did not respond to treatment. Unfortunately, one patient died from an overdose of illicit drugs, not with prescribed medication and one was admitted, and one felt worse, and one did not have any response.

However, 22 out of 50 patients were prescribed antipsychotic medication.11 out of 22 patients had ECG and blood done by the Crisis Team and 4 done by other parties (hospital and primary care).3 had recent blood tests but no ECG.2 patients did not have physical health monitoring and the reason was not documented.2 patients were started on antipsychotic by the Crisis Team, but the dose was not changed.

In terms of side effects, 8 out of 50 reported some side effects.

6 of them were prescribed antidepressants. They reported difficulty in sleeping and palpitations with Venlafaxine, nausea with Fluoxetine, nonspecific side effects with Citalopram, and sedation with Trazadone. 2 patients felt dizziness, diarrhoea, and muscle spasms with Mirtazapine. One patient had a metallic taste with Zopiclone. For side effects with antipsychotics, only one patient reported side effects with Olanzapine.

**Conclusion:**

- The Crisis Team is working at excellent standards on most areas of psychotropic prescribing and monitoring
- The Crisis Team needs to improve physical health monitoring of their patients.

